# Supplementary material for: Prophylactic and long-lasting efficacy of senolytic CAR T cells against age-related metabolic dysfunction
Source: Nat Aging. 2024 Jan 24;4(3):336–49. doi: 10.1038/s43587-023-00560-5 (PMC10950785; doi:10.1038/s43587-023-00560-5)
Supplement: Supplementary file 1 — Legends of Extended Data Figs. 1–10. [file 43587_2023_560_MOESM1_ESM.pdf]

nature aging

Article https://doi.org/10.1038/s43587-023-00560-5

Prophylactic and long-lasting efficacy of senolytic CAR T cells against age-related metabolic dysfunction

In the format provided by the authors and unedited

Extended Data Figure Legends

Extended Data Figure 1 | Characterization of uPAR-positive cells in aging. a, RNA expression of *Plaur* in liver, adipose tissue (fat) and muscle of young (3 months) or old (21 months) mice. Data obtained from the Tabula Muris Senis project.[18] **b**, Quantification of immunohistochemical staining of mouse uPAR in liver, adipose tissue, muscle and pancreas from young (age 3 months) or old (age 20 months) mice (n=3 per age). **c,** Hematoxylin and eosin staining and immunofluorescence staining of young (age 3 months n=3 mice) or old (age 18-20 months n=3 mice) livers. uPAR (green), β-gal (red), F4/80 (white), DAPI (blue). **d**, Percentage of SA-b-gal positive cells in young and aged livers in c. **e**, Hematoxylin and eosin staining and immunofluorescence staining of young (age 3 months n=3 mice) or old (age 18-20 months n=3 mice) pancreas. uPAR (green), β-gal (red), F4/80 (white), DAPI (blue). **f**, Percentage of SA-b-gal positive cells in young and aged livers in e. **g,h**, Correlation (Pearson's R value) of β-gal and F4/80 co-staining, β-gal and uPAR co-staining or uPAR and F4/80 co-staining in aged livers (**g**) and aged pancreas (**h**). **i,j**, Percentage of β-gal positive cells that costain for F4/80, uPAR or uPAR and F4/80 in aged livers (**i**) and aged pancreas (**j**). Data are mean ± s.e.m **(a,b,d,f-h);** values are derived from two-tailed unpaired Student's t-tests **(a,b,d,f)** one-way ANOVA with multiple comparisons **(g,h).** Results are from 1 independent experiment **(a-j)**.

Extended Data Figure 2 | Single cell profile of aged tissues. a, Dot plot showing expression of 34 signature genes across the 12 lineages of the liver. The size of the dots represents the proportion of cells expressing a particular marker, and the color scale indicates the mean expression levels of the markers (z-score transformed). **b,** Fractions of uPAR-positive and uPAR-negative cells in the various lineages in liver (n= the sequencing of 4 mice where 2 females were combined into one replicate and 2 males were combined into another replicate). Error bars represent s.d. **c,** Dot plot showing expression of 40 signature gene expressions across the 13 lineages of the adipose tissue. The size of the dots represents the proportion of cells expressing

a particular marker, and the color scale indicates the mean expression levels of the markers (z-score transformed). **d,** Fractions of uPAR-positive and uPAR-negative cells in the various lineages in adipose tissue (n= the sequencing of 4 mice where 2 females were combined into one replicate and 2 males were combined into another replicate). Error bars represent s.d. **e,** Dot plot showing expression of 40 genes across the 12 lineages of the pancreas. The size of the dots represents the proportion of cells expressing a particular marker, and the color scale indicates the mean expression levels of the markers (z-score transformed). **f,** Fractions of uPAR-positive and uPAR-negative cells in the various lineages in pancreas (n= the sequencing of 4 mice where 2 females were combined into one replicate and 2 males were combined into another replicate). Error bars represent s.d. Data are mean ± s.d.; p values are derived from two-tailed unpaired Student's t-tests **(b,d,f).** Results are from 1 independent experiment **(a-f)**.

Extended Data Figure 3 | Characteristics of senescent uPAR-positive cells in aged tissues. a-c, Molecular Signature Database Hallmark 2020 signatures that are significantly enriched in uPAR positive cells vs uPAR negative cells of liver **(a),** adipose tissue **(b)** and pancreas **(c). d-f,** quantification of the proportion of uPAR positive and negative cells by cell type contributing to the respective senescence signature in Fig. 1h **(d)**, Fig. 1j **(e)** and Fig. 1l **(f)**. **g-o,** UMAP visualizations with senescence signature scores[17] in each cell indicated by the color scale. Below: quantification of the proportion of uPAR positive and negative cells contributing to the respective senescence signature in total **(h,k,n)** and by cell type **(i,l,o). g,h,i,** liver; **j,k,l,** adipose tissue; **m,n,o;** pancreas. Results are from 1 independent experiment with (n= the sequencing of 4 mice where 2 females were combined into one replicate and 2 males were combined into another replicate) **(a-m)**.

Extended Data Figure 4 | Effect of uPAR CAR T cells on aged tissues. a-c, Quantification of SA-β-Gal–positive cells in adipose tissue, liver and pancreas 20 days after cell infusion (n=3 for UT; n=3 for h.19-m.28z; n=4 for m.uPAR-m.28z). **d-f,** Quantification of uPAR-positive cells in adipose tissue, liver and pancreas 20 days after cell infusion (n=3 per group). **g-j,** Percentage of

dendritic cells and uPAR$^+$ dendritic cells in the adipose tissue **(g,h)** or liver **(i,j)** 20 days after cell infusion (n=3 for UT; n=3 for h.19-m.28z; n=4 for m.uPAR-m.28z). **k-n,** Percentage of macrophages and uPAR$^+$ macrophages in the adipose tissue **(k,l,)** or liver **(m,n)** 20 days after cell infusion (n=3 for UT; n=3 for h.19-m.28z; n=4 for m.uPAR-m.28z). **o-r,** Percentage of monocytes and uPAR$^+$ monocytes in the adipose tissue **(o,p)** or liver **(q,r)** 20 days after cell infusion (n=3 for UT; n=3 for h.19-m.28z; n=4 for m.uPAR-m.28z). Results of 1 independent experiment **(a-r)**. Data are mean ± s.e.m.; p values from two-tailed unpaired Student's t-test **(a-r)**.

Extended Data Figure 5 | uPAR CAR T cells are not associated with signs of tissue damage in aged tissues and do not exacerbate spontaneous age-related histological changes in lung, liver and kidneys. Mice received cell infusions at 18-20 months and were sacrificed 20 days after infusion of the indicated T cells. Sections were stained with hematoxylin and eosin. Aged mice showed mononuclear leukocytic aggregates composed predominantly of lymphocytes and plasma cells in tissues in an age dependent manner. These leukocytic aggregates were more frequently observed in tissues from uPAR-m.28z CAR T- treated aged mice than tissues from control aged mice and were not associated with necrosis and/or degeneration in tissues from both experimental and control aged mice. These lymphocytic and plasmocytic aggregates in tissues are often observed in naïve aged mice and are considered spontaneous background findings in longitudinal aging studies in mice[50,51]. **a**, Representative sections of normal cerebral cortex and meninges at the level of the posterior hypothalamus (inset: hippocampus). **b**. Histology of normal cardiomyocytes and interstitium in myocardium (inset: ventricles and interventricular septum). **c**. Representative histology of normal lungs showed dense aggregates of lymphocytes and fewer plasma cells and macrophages around bronchioles or vasculature (inset: pulmonary lobes). **d**. The liver from aged mice showed accumulation of lymphocytic and histiocytic aggregates in portal to periportal regions (Inset: hepatic lobe). **e**. Histology of the kidneys showed accumulation of lymphocytes and plasma cells in the renal interstitium (n & o) and around blood vessels (inset:

renal cortex, medulla, and pelvis). **f**. Representative sections of normal pancreatic acini (exocrine pancreas) and islets of Langerhans (endocrine pancreas; inset: pancreatic lobule). Images were captured at 4x (insets) and 40x magnifications. Results of 1 independent experiment (with n=3 per group).

Extended Data Figure 6 | Effect of uPAR CAR T cells in young and old tissues. a-b, Mice received cell infusion at 3 months old. **a,** Levels of glucose before (0 min) and after intraperitoneal administration of glucose (2 g/kg) 2.5 months after cell infusion (n=13 for untransduced T cells; n=12 for h.19-m.28z and n=13 for m.uPAR-m.28z). **b,** Area under the curve (AUC) representing the results from **a**. Each point represents a single mouse. **c-d,** Mice received cell infusion at 18-20 months old. **c,** Levels of glucose before (0 min) and after intraperitoneal administration of insulin (0.5 units/kg body weight) 2.5 months after cell infusion (n=10 for untransduced T cells and n=10 for m.uPAR-m.28z). **d,** Area under the curve (AUC) representing the results from **c**. Each point represents a single mouse. Results of 2 independent experiments **(a,b)** or 1 independent experiment **(c,d)**. Data are mean ± s.e.m.; p values from two-tailed unpaired Student's t-test **(b,d)**.

Extended Data Figure 7 | Profile of and long-term effects of uPAR CAR T cells in aging. a,b, Percentage of $CD4^+$ or $CD8^+$ cells among $CD45.1^+$ T cells from the spleen (**a**) or liver (**b**) of 4-month-old or 20-month-old mice 20 days after cell infusion (n=3 mice per age group for untransduced T cells [UT] and for h.19-m.28z; n=4 for m.uPAR-m.28z). **c,d,** Percentage of $CD45.1^+$ T cells expressing differentiation markers CD62L and CD44 in the spleen (**c**) or liver (**d**) of 4-month-old or 20-month-old mice 20 days after cell infusion (sample sizes as in **a**). **e,f,** Percentage of $CD4^+$ or $CD8^+$ cells among $CD45.1^+$ T cells in the spleen (**e**) or liver (**f**) of 15-month-old mice 12 months after cell infusion (n=3 mice per group). **g,h,** Percentage of $CD45.1^+$ T cells expressing differentiation markers CD62L and CD44 on $CD45.1^+$ T cells in the spleen (**g**) or liver (**h**) of 15-month-old mice 12 months after cell infusion (n=3 mice per group). **i,** Time to exhaustion

in exercise capacity testing 12 months after cell infusion (n=8 for untransduced T cells; n=6 for h.19-m.28z; n=12 for m.uPAR-m.28z). **j,** Maximum speed (m/min) in capacity testing 12 months after cell infusion (sample sizes as in **i**). **k-m,** Quantification of SA-β-Gal–positive cells 12 months after cell infusion in **(k)** adipose tissue (n=6 for UT; n=5 for h.19-m.28z; n=6 for m.uPAR-m.28z); **(l)** liver (n=6 for UT; n=5 for h.19-m.28z; n=5 for m.uPAR-m.28z) and **(m)** pancreas (n=6 for UT; n=5 for h.19-m.28z; n=6 for m.uPAR-m.28z). **n-p,** Quantification of uPAR-positive cells in **(n)** adipose tissue, **(o)** liver and **(p)** pancreas 12 months after cell infusion (n=3 per group). Results of 1 independent experiment **(a-h, n-p)** or 2 independent experiments **(i-m)**. Data are mean ± s.e.m.; p values from two-tailed unpaired Student's t-test **(a-h, k-p)** or two-tailed Mann Whitney test **(i,j)**.

Extended Data Figure 8 | uPAR CAR T cells decrease senescent cell burden in therapeutic and preventive settings in high fat diet. a, Representative staining of SA-β-Gal after two months of high fat diet or normal chow diet. **b-d;** Quantification of SA-β-Gal–positive cells in pancreas, liver and adipose tissue after two months of high fat diet or normal chow diet (n=3 for chow; n=3 HFD). **e,** Representative staining of SA-β-Gal 1 month after cell infusion in the experimental scheme depicted in Fig. 7a. **f-h;** Quantification of SA-β-Gal–positive cells in pancreas, liver and adipose tissue 1 month after cell infusion (n=5 for UT; for m.uPAR-m.28z n=5 in pancreas, n=6 in liver and n=3 in adipose tissue). UT, untransduced T cells. **i,** Representative staining of SA-β-Gal 3.5 months after cell infusion in the experimental scheme depicted in Fig. 7j. **j-l,** Quantification of SA-β-Gal–positive cells in pancreas, liver, and adipose tissue 3.5 months after cell infusion (UT n=4 in pancreas, n=5 in liver and adipose tissue; for m.uPAR-m.28z n=5). Each panel shows results from 1 experiment. Data are mean ± s.e.m.; p values from two-tailed unpaired Student's t-test **(b-d; f-h; j-l).**

Extended Data Figure 9 | Profile and persistence of uPAR CAR T cells in metabolic syndrome. T cells were assessed in spleen (**a-d**) and liver (**e-h**) 3.5 months after cell infusion in the experimental scheme depicted in Fig. 7j. **a,** Percentage of $CD45.1^+$ T cells in the spleen. **b,** Percentage of $CD4^+$ cells among $CD45.1^+$ T cells in the spleen. **c,** Percentage of $CD8^+$ cells among $CD45.1^+$ T cells in the spleen. **d,** Percentage of $CD45.1^+$ T cells from the spleen expressing differentiation markers CD62L and CD44. **e,** Percentage of $CD45.1^+$ T cells in the liver. **f,** Percentage of $CD4^+$ cells among $CD45.1^+$ T cells in the liver. **g,** Percentage of $CD8^+$ cells among $CD45.1^+$ T cells in the liver. **h,** Percentage of $CD45.1^+$ T cells in the liver expressing differentiation markers CD62L and CD44. Results in each panel are from 1 experiment (n=5 mice per group). Data are mean $\pm$ s.e.m.; p values from two-tailed unpaired Student's t-test.

Extended Data Figure 10 | Gating strategies. a,b, Representative flow cytometry staining of m.uPAR-m.28z **(a)** or untransduced T cells **(b)** obtained from the spleens of mice 20 days after cell infusion as depicted in Fig. 5g. Shown are results of 1 independent experiment (n=3 mice for untransduced T cells; n=4 mice for m.uPAR-m.28z).
