# Supplementary material for: Prophylactic and long-lasting efficacy of senolytic CAR T cells against age-related metabolic dysfunction
Source: Nat Aging. 2024 Jan 24;4(3):336–49. doi: 10.1038/s43587-023-00560-5 (PMC10950785; doi:10.1038/s43587-023-00560-5)
Supplement: Supplementary file 2 — Reporting Summary [file 43587_2023_560_MOESM2_ESM.pdf]

nature portfolio

Corresponding author(s): Corina Amor Vegas

Last updated by author(s): Dec 11, 2023

Reporting Summary

Nature Portfolio wishes to improve the reproducibility of the work that we publish. This form provides structure for consistency and transparency in reporting. For further information on Nature Portfolio policies, see our Editorial Policies and the Editorial Policy Checklist.

Statistics

For all statistical analyses, confirm that the following items are present in the figure legend, table legend, main text, or Methods section.

n/a	Confirmed	
☐	☒	The exact sample size (*n*) for each experimental group/condition, given as a discrete number and unit of measurement
☐	☒	A statement on whether measurements were taken from distinct samples or whether the same sample was measured repeatedly
☐	☒	The statistical test(s) used AND whether they are one- or two-sided<br>*Only common tests should be described solely by name; describe more complex techniques in the Methods section.*
☒	☐	A description of all covariates tested
☐	☒	A description of any assumptions or corrections, such as tests of normality and adjustment for multiple comparisons
☐	☒	A full description of the statistical parameters including central tendency (e.g. means) or other basic estimates (e.g. regression coefficient) AND variation (e.g. standard deviation) or associated estimates of uncertainty (e.g. confidence intervals)
☐	☒	For null hypothesis testing, the test statistic (e.g. *F*, *t*, *r*) with confidence intervals, effect sizes, degrees of freedom and *P* value noted<br>*Give P values as exact values whenever suitable.*
☒	☐	For Bayesian analysis, information on the choice of priors and Markov chain Monte Carlo settings
☒	☐	For hierarchical and complex designs, identification of the appropriate level for tests and full reporting of outcomes
☐	☒	Estimates of effect sizes (e.g. Cohen's *d*, Pearson's *r*), indicating how they were calculated

Our web collection on statistics for biologists contains articles on many of the points above.

Software and code

Policy information about availability of computer code

Data collection	BD-Fortessa cytometer, SONY SH800S sorter, ZEISS Axio Observer microscopy, BX45 microscope, 10X Genomics' Cell Ranger software v7.1.0, IDEXX Procyte DX, Beckman Coulter AU680 analyzer, Microsoft Excel for Mac.
Data analysis	FlowJo 10.8.1, GraphPad Prism V.9.3.1, Fiji-ImageJ v2.1.051

For manuscripts utilizing custom algorithms or software that are central to the research but not yet described in published literature, software must be made available to editors and reviewers. We strongly encourage code deposition in a community repository (e.g. GitHub). See the Nature Portfolio guidelines for submitting code & software for further information.

Data

Policy information about availability of data

All manuscripts must include a data availability statement. This statement should provide the following information, where applicable:

- Accession codes, unique identifiers, or web links for publicly available datasets
- A description of any restrictions on data availability
- For clinical datasets or third party data, please ensure that the statement adheres to our policy

Source data is provided with this paper. scRNA-seq data presented in this study is deposited in the Gene Expression Omnibus (GEO) database under accession number GSE243616. Data from the Tabula Muris Senis project18 was accessed through https://twc-stanford.shinyapps.io/maca/. Human dataset from26 was

accessed through https://zenodo.org/records/7311202#.Y20ybezMIyl. Requests for materials should be addressed to the corresponding authors.
All code is available at https://github.com/naikai/Amor_et_al_2023

Human research participants

Policy information about studies involving human research participants and Sex and Gender in Research.

Reporting on sex and gender	Not applicable
Population characteristics	Not applicable
Recruitment	Not applicable
Ethics oversight	Not applicable

Note that full information on the approval of the study protocol must also be provided in the manuscript.

Field-specific reporting

Please select the one below that is the best fit for your research. If you are not sure, read the appropriate sections before making your selection.

☒ Life sciences ☐ Behavioural & social sciences ☐ Ecological, evolutionary & environmental sciences

For a reference copy of the document with all sections, see nature.com/documents/nr-reporting-summary-flat.pdf

Life sciences study design

All studies must disclose on these points even when the disclosure is negative.

Sample size	No statistical methods were used to pre-determine sample size. Sample sizes were estimated based on preliminary experiments, with an effort to achieve a minimum of n=3 mice per treatment group which proved to be sufficient to reproducibly observe a statistical significant difference. For statistical testing we did not include adjustment for multiple comparisons. The rationale for this is that , two control groups were compared to the experimental group, but it could have been biologically possible to just have one control group. Thus, for any given endpoint, there were two pairwise comparisons: the experiment group separately compared to each control. While two tests were evaluated, we only considered the analysis statistically significant if both tests had a p-value less than 0.05. If only one of the two tests was significant, we did not claim the groups were significantly different; instead, we considered the analysis inconclusive and reported a trend. Viewing the analysis as significant only if both p-values were less than 0.05 preserves the family-wise error rate at less than 0.05. Therefore, there is no need to further adjust for multiple comparisons.
Data exclusions	No data was excluded. For histological assesment of HFD experiments OCT cassettes of samples containing adipose tissue or pancreas that were folded and presented a morphology that did not allowed for successful slide generation were not further processed.
Replication	All experiments were repeated in replicates and/or from different subjects in independent experiments. All attempts at replication were successful. Information for replication of each experiment is included in the figure legends.
Randomization	Mice were randomly assigned into treatment groups ensuring equal sex distribution among groups.
Blinding	Mouse conditions were observed by an operator who was blinded to the treatment groups in addition to the main investigator who was not blind to group allocation. Pathological analysis and exercise testing studies were performed in a blinded fashion. Data analysis was not performed in a blinded fashion. Data analysis are based on objectively measurable data (cell count, blood tests).

Reporting for specific materials, systems and methods

We require information from authors about some types of materials, experimental systems and methods used in many studies. Here, indicate whether each material, system or method listed is relevant to your study. If you are not sure if a list item applies to your research, read the appropriate section before selecting a response.

Materials & experimental systems

n/a	Involved in the study
☐	☒ Antibodies
☒	☐ Eukaryotic cell lines
☒	☐ Palaeontology and archaeology
☐	☒ Animals and other organisms
☒	☐ Clinical data
☒	☐ Dual use research of concern

Methods

n/a	Involved in the study
☒	☐ ChIP-seq
☐	☒ Flow cytometry
☒	☐ MRI-based neuroimaging

Antibodies

Antibodies used

The following fluorophore-conjugated antibodies were used in the indicated dilutions: Myc-tag AF647 (clone 9B11, Cell Signaling Technology, 2233S, lot 25, 1:50), m.CD45.1 BV785 (clone A20, Biolegend, 110743, lot B347719, 1:100), m.CD45.2 BV785 (clone 104, Biolegend, 109839, lot B343292, 1:100), mCD3 AF488 (clone 17A2, Biolegend, 100210, lot B284975, 1:100), mCD4 BUV395 (clone GK1.5, BD, 563790, lot 1097734, 1:50), mCD8 PE-Cy7 (clone 53-6.7, Biolegend, 100722, lot B312604, 1:50), mCD62L BV421 (clone MEL-14, Biolegend, 104435, lot B283191, 1:50), mCD44 APC Cy7 (clone IM7, BD Pharminogen, 560568, lot 1083068, 1:100), mCD3 BV650 (clone 17A2, Biolegend, 100229, lot B350667, 1:100),mCD19 BV650 (clone1D3, BD Biosciences, 563235, lot 1354015 1:100), mNKp46 BV650 (clone 29A1.4, Biolegend, 137635, lot B298809 1:100), mCD11b BUV395 (clone M1/70, BD Biosciences, 563553, lot 0030272 1:50), mLy-6C APC-Cy7 (clone HK1.4, Biolegend, 128026, lot B375238 1:100), mly6G BV605 (clone 1A8, BD Biosciences, 563005, lot2144780 1:100), m.uPAR AF700 (R&D systems, FAB531N, lot 1656339, 1:50), m.uPAR PE (R&D systems, FAB531P, lot ABLH0722051, 1:50), mF4/80 PE-eFluor610 (Clone BM8, Invitrogen, 61-4801-82, lot 2338698, 1:100). uPAR (AF534,R&D lot DCL0521042, 1:50), F4/80 (CI:A3-1, BioRad, Lot 155529, 1:100), anti-Horse anti-goat IgG (30116; Vector Laboratories, lot ZH0526). 7-AAD (BD, 559925, lot 9031655, 1:40) or Ghost UV 450 Viability Dye (13-0868-T100, Tonbo Biosciences lot D0868083018133, 1ul/ml) was used as viability dye.

Validation

All used antibodies were titrated. All the antibodies are validated for use in flow cytometry or immunohistochemistry or immunofluorescence. Data are available at the manufacturer's website. All used antibodies are commercially available. The following antibodies have been validated by the manufacturer against the mentioned species: Myc-tag AF647 (clone 9B11, Cell Signaling Technology, 2233S, lot 25, 1:50 all species), m.CD45.1 BV785 (clone A20, Biolegend, 110743, lot B347719, 1:100, mouse), m.CD45.2 BV785 (clone 104, Biolegend, 109839, lot B343292, 1:100, mouse), mCD3 AF488 (clone 17A2, Biolegend, 100210, lot B284975, 1:100, mouse), mCD4 BUV395 (clone GK1.5, BD, 563790, lot 1097734, 1:50, mouse), mCD8 PE-Cy7 (clone 53-6.7, Biolegend, 100722, lot B312604, 1:50, mouse), mCD62L BV421 (clone MEL-14, Biolegend, 104435, lot B283191, 1:50, mouse), mCD44 APC Cy7 (clone IM7, BD Pharminogen, 560568, lot 1083068, 1:100, mouse), mCD3 BV650 (clone 17A2, Biolegend, 100229, lot B350667, 1:100,mouse),mCD19 BV650 (clone1D3, BD Biosciences, 563235, lot 1354015 1:100,mouse), mNKp46 BV650 (clone 29A1.4, Biolegend, 137635, lot B298809 1:100,mouse), mCD11b BUV395 (clone M1/70, BD Biosciences, 563553, lot 0030272 1:50,mouse), mLy-6C APC-Cy7 (clone HK1.4, Biolegend, 128026, lot B375238 1:100,mouse), mly6G BV605 (clone 1A8, BD Biosciences, 563005, lot2144780 1:100,mouse), m.uPAR AF700 (R&D systems, FAB531N, lot 1656339, 1:50, mouse), m.uPAR PE (R&D systems, FAB531P, lot ABLH0722051, 1:50,mouse), mF4/80 PE-eFluor610 (Clone BM8, Invitrogen, 61-4801-82, lot 2338698, 1:100,mouse). uPAR (AF534,R&D lot DCL0521042, 1:50,mouse), F4/80 (CI:A3-1, BioRad, Lot 155529, 1:100,mouse), anti-Horse anti-goat IgG (30116; Vector Laboratories, lot ZH0526, mouse).

Animals and other research organisms

Policy information about studies involving animals; ARRIVE guidelines recommended for reporting animal research, and Sex and Gender in Research

Laboratory animals

The following mice were used: 3- to 4-month-old C57BL/6 mice (purchased from Charles River), 18-20-month-old C57BL/6 mice (obtained from the National Institute of Aging or the Jackson Laboratory), and 6-week-old B6.SJL-Ptrca/BoyAiTac (CD45.1 mice) (purchased from Taconic). Mice of both sexes were used at 8-12 weeks of age and 18-20 months of age for the aging experiment, males of 8-12 weeks for the high fat diet experiments and females of 6-10 weeks old for T cell isolation. Mice were maintained under specific pathogen-free conditions. Housing was on a 12h-12h light-dark cycle under standard temperature and humidity of approximately 18-24C and 40-60%, respectively. Mice were kept in group housing. Mice had free access to food and water except during the starvation period before glucose or insulin tolerance testing. Aging mice were fed a normal diet (PicoLab Rodent Diet 20, LabDiet), mice on the high fat diet (HFD) experiments were fed a HFD (TD.06414, 60% of kcal from fat; Envigo).

Wild animals

This study did not involve wild animals.

Reporting on sex

Mice of both sexes were used at 8-12 weeks of age and 18-20 months of age for the aging experiment, males of 8-12 weeks for the high fat diet experiments and females of 6-10 weeks old for T cell isolation.

Field-collected samples

This study did not involve samples collected from the field.

Ethics oversight

Memorial Sloan Kettering Cancer Center (MSKCC) and Cold Spring Harbor Laboratory (CSHL) Internal Animal Care and Use Committee (animal protocol 11-06-011 at MSKCC and 21-4 at CSHL).

Note that full information on the approval of the study protocol must also be provided in the manuscript.

Flow Cytometry

Plots

Confirm that:

☒ The axis labels state the marker and fluorochrome used (e.g. CD4-FITC).

☒ The axis scales are clearly visible. Include numbers along axes only for bottom left plot of group (a 'group' is an analysis of identical markers).

☒ All plots are contour plots with outliers or pseudocolor plots.

☒ A numerical value for number of cells or percentage (with statistics) is provided.

Methodology

Sample preparation	Livers were dissociated using the MACS liver dissociation kit (Miltenyi Biotec, 130-1-5-807), filtered through a 100-µm strainer and washed with PBS, and red blood cells were lysed by an ACK (ammonium–chloride–potassium) lysing buffer (Lonza). Cells were washed with PBS, resuspended in FACS buffer and either used for immediate analysis or fixed with Fixation Buffer (BD Biosciences; 554655) according to the manufacturer's instructions and used for later analysis. Spleens were mechanically disrupted with the back of a 5-ml syringe, filtered through a 40-µm strainer and washed with PBS and 2 mM EDTA, then red blood cells were lysed by ACK lysing buffer (Lonza). Cells were washed with PBS and 2 mM EDTA, resuspended in FACS buffer and either used for immediate analysis or fixed with Fixation Buffer (BD Biosciences;554655) and used for later analysis. Gonadal adipose tissue was dissociated as described43. In short, adipose tissue was isolated and placed in a digestion solution consisting of 4 mg/ml collagenase, type II (Sigma) in DPBS (Life Technologies) supplemented with 0.5% BSA (Sigma) and 10 mM CaCl2 digested at 37° C for 20 min in a rotational shaker (200 rpm). Afterwards, samples were mechanically dissociated with a 10-ml serological pipette, filtered through a 40-µm strainer and washed with PBS and 2 mM EDTA, then red blood cells were lysed by ACK lysing buffer (Lonza). Pancreata were placed into cold DMEM with 10% FBS and penicillin and streptomycin. The pancreata were minced in this media on ice into 2- to 4-mm fragments so that they would pass through the end of 1-ml pipette tip with ease. The minced tissue was collected in a 15-ml Falcon tube and dissociated in 100 mg/ml Dispase (Life Tech., cat. 17105041), 20 mg/ml collagenase P (Roche, cat. 11249002001) and 1 mM EDTA for 20 minutes on a heated rocker at 37° C (Eppendorf). After 20 minutes, 5 ml of DMEM with 10% FBS was added to quench the reaction. The supernatant was removed and filtered through a 100-µm filter(VWR). Next, 5 ml of dissociation media consisting of 100 mg/ml Dispase (Life Tech., cat. 17105041), 20 mg/ml collagenase P (Roche, cat. 11249002001) and 1 mM EDTA was added prior to replacing the 15-ml tube into the heated rocker for 20 minutes. The reaction was quenched again after 20 minutes with media and filtered via a 100-µm filter. The dissociated cells were spun at 500 rcf for 10 minutes in a swinging bucket centrifuge. The supernatant was discarded and the cells were resuspended in ACK lysis buffer for 2-4 minutes in ice. Cells were washed with PBS, resuspended in FACS buffer and either used for immediate analysis or fixed with Fixation Buffer (BD Biosciences;554655) and used for later analysis.
Instrument	Fortessa 3,BD.
Software	Collection: FACS DIVA.<br>Analysis: Flowjo 10.1
Cell population abundance	The purity was verified by flow cytometry.
Gating strategy	The starting cell population was gated on a SSC-A/FSC-A plot. Cell siglets were identified by FSC/SSC gating. Positive/Negative populations were determined by FMO controls.

☒ Tick this box to confirm that a figure exemplifying the gating strategy is provided in the Supplementary Information.

March 2021
